# Viperin mutation is linked to immunity, immune cell dynamics, and metabolic alteration during VHSV infection in zebrafish

**DOI:** 10.3389/fimmu.2023.1327749

**Published:** 2023-12-19

**Authors:** K. A. S. N. Shanaka, Sumi Jung, K. P. Madushani, Myoung-Jin Kim, Jehee Lee

**Affiliations:** ^1^ Department of Marine Life Sciences & Fish Vaccine Research Center, Jeju National University, Jeju, Republic of Korea; ^2^ Marine Science Institute, Jeju National University, Jeju, Republic of Korea; ^3^ Bioresource Industrialization Center, Nakdonggang National Institute of Biological Resources, Sangju-si, Gyeongsangbuk-do, Republic of Korea

**Keywords:** VHSV, RNA virus, Viperin, zebrafish, antiviral protein

## Abstract

Viperin is a prominent antiviral protein found in animals. The primary function of Viperin is the production of 3’-deoxy-3’,4’-didehydro-cytidine triphosphate (ddhCTP), an inhibitory nucleotide involved in viral RNA synthesis. Studies in mammalian models have suggested that ddhCTP interferes with metabolic proteins. However, this hypothesis has yet to be tested in teleost. In this study, the role of Viperin in regulating metabolic alterations during viral hemorrhagic septicemia virus (VHSV) infection was tested. When infected with VHSV, *viperin* ^-/-^ fish showed considerably higher mortality rates. VHSV copy number and the expression of the *NP* gene were significantly increased in *viperin* ^-/-^ fish. Metabolic gene analysis revealed significant differences in *soda*, *hif1a*, *fasn*, and *acc* expression, indicating their impact on metabolism. Cholesterol analysis in zebrafish larvae during VHSV infection showed significant upregulation of cholesterol production without Viperin. *In vitro* analysis of ZF4 cells suggested a considerable reduction in lipid production and a significant upregulation of reactive oxygen species (ROS) generation with the overexpression of *viperin*. Neutrophil and macrophage recruitment were significantly modulated in *viperin* ^-/-^ fish compared to the wild-type (WT) fish. Thus, we have demonstrated that Viperin plays a role in interfering with metabolic alterations during VHSV infection.

## 1 Introduction

Viral infections alter cellular metabolism as a part of host–pathogen interactions. Hijacking glucose metabolism by viruses, such as by upregulating Glut transporter expression, is necessary to foster viral life cycle events and viral biomass accumulation. In terms of host side of infections, ATP produced by aerobic glycolysis is required for interferon-gamma (IFN-γ) responses. In immune cells, including macrophages and neutrophils, reactive oxygen species (ROS) are necessary to upgrade their inflammatory status. Therefore, the proper balance of virus-induced vs. host cellular metabolism during viral infection determines the course of the disease (1, 2).

Viperin is a virus-induced antiviral protein, and growing evidence is suggesting a role of Viperin during virus-mediated metabolic alterations (3). The metabolic regulation in which Viperin participates is unique, and no other antiviral protein known to date can replace the mechanism of Viperin (4). The product of Viperin, 3’-deoxy-3’,4’-didehydro-cytidine triphosphate (ddhCTP), lacks the 3′-OH group required for RNA chain elongation; thus, the direct role of this molecule is to participate in viral RNA replication termination. Additionally, ddhCTP has been shown to bind to the nicotinamide adenine dinucleotide (NAD)+-binding pocket of metabolic enzymes, including glyceraldehyde 3-phosphate dehydrogenase (GAPDH) and lactate dehydrogenase (LDH), to reduce their catalytic activities (5). This binding may alter metabolic pathways. Metabolic alterations may interfere with immune cell dynamics, as immune cells are driven by metabolism, such as *via* Nicotinamide adenine dinucleotide phosphate (NADPH) to generate ROS for proper recruitment and activation during viral infections. Therefore, *viperin* ^-/-^ cells may interfere with immune cell activity.

Viral infections can alter the cell membrane composition, enhance cholesterol and triglyceride levels (6), and facilitate viral egress. Several studies have demonstrated the ability of Viperin to alter the cholesterol ratio in cell membranes (3, 7). ddhCTP has been shown to inhibit the cholesterol biosynthesis pathway directly, and reduced cholesterol in cell membranes may be negatively associated with the release of enveloped viruses *via* budding.

Viral hemorrhagic septicemia virus (VHSV) is a negative-strand virus of the genus *Novirhabdovirus* (8, 9). Although the importance of cholesterol in VHSV propagation has been established (10), the mechanism by which cholesterol modulation occurs in fish needs to be better defined. In a previously published paper, we described the successful generation of *viperin* ^-/-^ zebrafish, emphasizing the importance of Viperin in larval defense against viruses (11). In this study, we analyzed the effects of Viperin on macrophage and neutrophil migration patterns in *viperin* ^-/-^ fish and cellular metabolism. To our knowledge, no previous study has analyzed the metabolic alterations or immune cell migration caused by Viperin during VHSV infection in fish.

## 2 Materials and methods

### 2.1 Zebrafish husbandry

Wild-type (WT) and *viperin* ^-/-^ zebrafish were maintained as previously described (12). Water used in tanks was maintained at constant pH (pH 6.8–7.5), conductivity (500–800 μS), and temperature (28°C ± 0.5°C) during the fish rearing. The zebrafish facility’s light and dark cycle consisted of 14:10 h. The fish were fed three times with the Artemia diet. The Jeju National University Animal Ethics Committee reviewed and approved this study.

### 2.2 Mortality experiments

Three-month-old male zebrafish, including WT and the *viperin* ^-/-^ fish (11), were gradually brought to the experimental temperature of 15°C by reducing the temperature from 28°C (2°C reduced/day). The fish were acclimatized at 15°C for 1 week and evaluated for low-temperature adaptation, and those with abnormal phenotypes or behaviors were removed.

Virus culture and propagation were performed as previously described (11). Acclimatized fish were injected with two doses of WT VHSV (genotype 4a) (high amount = 5 × 10^6^ TCID_50_/fish and low dose =1 × 10^6^ TCID_50_/fish) intraperitoneally. A 10-µL injection needle (World Precision Instruments, FL, USA) was used for injection. Fish were anesthetized with tricaine (MS-222; 160 μg/mL) solution before the injection, and 5 µL of VHSV was administered. After the injection, the fish were carefully reviewed. Fish that died within the first 3 days were removed from the recording, and data acquiring was started from the fourth day.

### 2.3 VHSV challenge experiment

Three-month-old adult male zebrafish were used for the VHSV challenge experiments. The fish were adapted to the experimental temperature described in the mortality experiment and injected with 1 × 10^5^ TCID_50_/mL of VHSV. After the initial injection, the VHSV copy number was checked at 12 h postinfection (hpi) to evaluate the injection process. The internal tissues of the injected fish were sampled at 1, 3, 5, 7, and 9 Days post infection (dpi) and snap-frozen in liquid nitrogen.

### 2.4 RNA isolation and RT-qPCR

TRIzol reagent (Thermo Fisher Scientific, Waltham, MA, USA) was used for RNA extraction. Extracted RNA (3 µg) was used for cDNA synthesis using the PrimeScript 1st strand cDNA synthesis kit (Takara, Japan). The cDNA was diluted with nuclease-free water according to the Ct value of the reference gene (**Supplementary Table S1**). VHSV copy number was calculated based on the previously described method (13).

The qPCR primers for gene expression analysis were designed according to the guidelines for the publication of quantitative real-time PCR experiments (MIQE) experiments (14). The RT-qPCR Dice™ TP950 Real-Time Thermal Cycler System (Takara, Japan) was used to analyze the gene expression patterns.

### 2.5 Immune cell migration analysis

For neutrophil and macrophage analysis, the *Tg(mpx:mCherry)* and *Tg*(*mpeg:EGFP*) lines (15) were crossed with the *viperin* ^-/-^, and the *viperin* KO transgenic line was generated. To introduce a wound, the caudal fin of larvae at 5 days postfertilization (dpf) was amputated and immediately submerged in poly I:C solution (15 µg/mL). Immune cell recruitment to the wound area was analyzed using a fluorescence microscope (×40; Leica Microsystems, Germany).

### 2.6 Viperin-stable cell line preparation

The *viperin* coding sequence was PCR-amplified using primers (synthesized by Macrogen, Korea) designed to contain *Hind*III and *Xho*I restriction sites (**Supplementary Table S1**). The PCR products were separated on an agarose gel and purified using the gel purification method (Bioneer, Republic of Korea). pcDNA3.1+ vector (Invitrogen, USA) and the purified PCR products were digested using the *Hind*III and *Xho*I restriction enzymes (TaKaRa, Japan). The digested vector: the PCR products were mixed at a 1:3 ratio, and ligation was performed. The ligated products were transformed into a DH5α competent *Escherichia coli*. Positive clones were selected by colony PCR and confirmed by sequencing (Macrogen, Korea). The selected clones were expanded in Luria–Bertani (LB) growth medium (BD Difco) supplemented with penicillin. After overnight culture, the vector was harvested using a plasmid extraction kit (Qiagen, Hilden, Germany). For stable cell preparation, vectors were linearized with the *Bgl*II restriction enzyme (Takara, Japan) and transfected into ZF4 cells (CRL-2050, ATCC, USA). Positive cells were selected based on Geneticin (Thermo Fisher Scientific, USA) resistance, and these positive colonies were expanded in L-15 growth medium supplemented with fetal bovine serum (FBS) 10%, 1% penicillin-streptomycin, and 1 mg/mL gentamycin.

### 2.7 ROS analysis

Viperin-stable cells were treated with L-15 medium containing glucose 30 mM for 6 h at 28°C along with the respective controls for ROS stimulation. The supernatant was carefully removed after 6 h, and the cells were washed with plain L-15 medium. L-15 growth medium containing 10 µM 2′,7′-dichlorofluorescein diacetate (DCFH-DA, D6883, Merck, Germany) was added to the cell culture well, and the plate was incubated for 30 min. Fluorescence was quantified using a fluorometer (SYNERGY, HTX, BioTeK, USA). For the reference assay, total protein was quantified using the Bradford method (Bio-Rad, USA).

### 2.8 Cholesterol and lipid analysis

Ten larvae from each group were injected with VHSV (~10^3^ TCID_50_/larvae), and control fish were injected with phosphate-buffered saline (PBS). Fish were incubated at 18°C. At different time points, the fish were frozen and homogenized in 1 mL PBS, and cholesterol levels were measured using a Stanbio Cholesterol LiquiColor^®^ kit (Stanbio Laboratory, TX, USA). Cholesterol content was standardized according to the amount of genomic DNA in the samples.

Viperin-stabilized cells were used for *in vitro* lipid analysis. The cells were infected with rVHSV (16) and fixed in 4% paraformaldehyde (Electron Microscopy Sciences) at room temperature (RT) for 10 min. The cells were washed with 1× PBS. The working oil redo solution (Sigma, USA) was prepared by mixing the oil redo stock (0.5% in 2-propanol) in dH_2_O at a 3:2 ratio. The working solution was added to the cells, which were subsequently incubated for 15 min at RT. The cells were washed with dH_2_O and imaged at ×40 magnification (Leica Microsystems, Germany).

### 2.9 Statistical analysis

Data were analyzed using analysis of variance with Tukey’s *post-hoc* test. Different lowercase letters indicate statistical significance (*p* < 0.05). The Kaplan–Meier method was used for survival analysis, and the statistical significance of the survival data was analyzed using the Mantel–Cox test (*p* < 0.05).

## 3 Results

### 3.1 Mortality assay, immune challenge experiment, VHSV copy number, and *NP* gene expression

**Figure 1:**
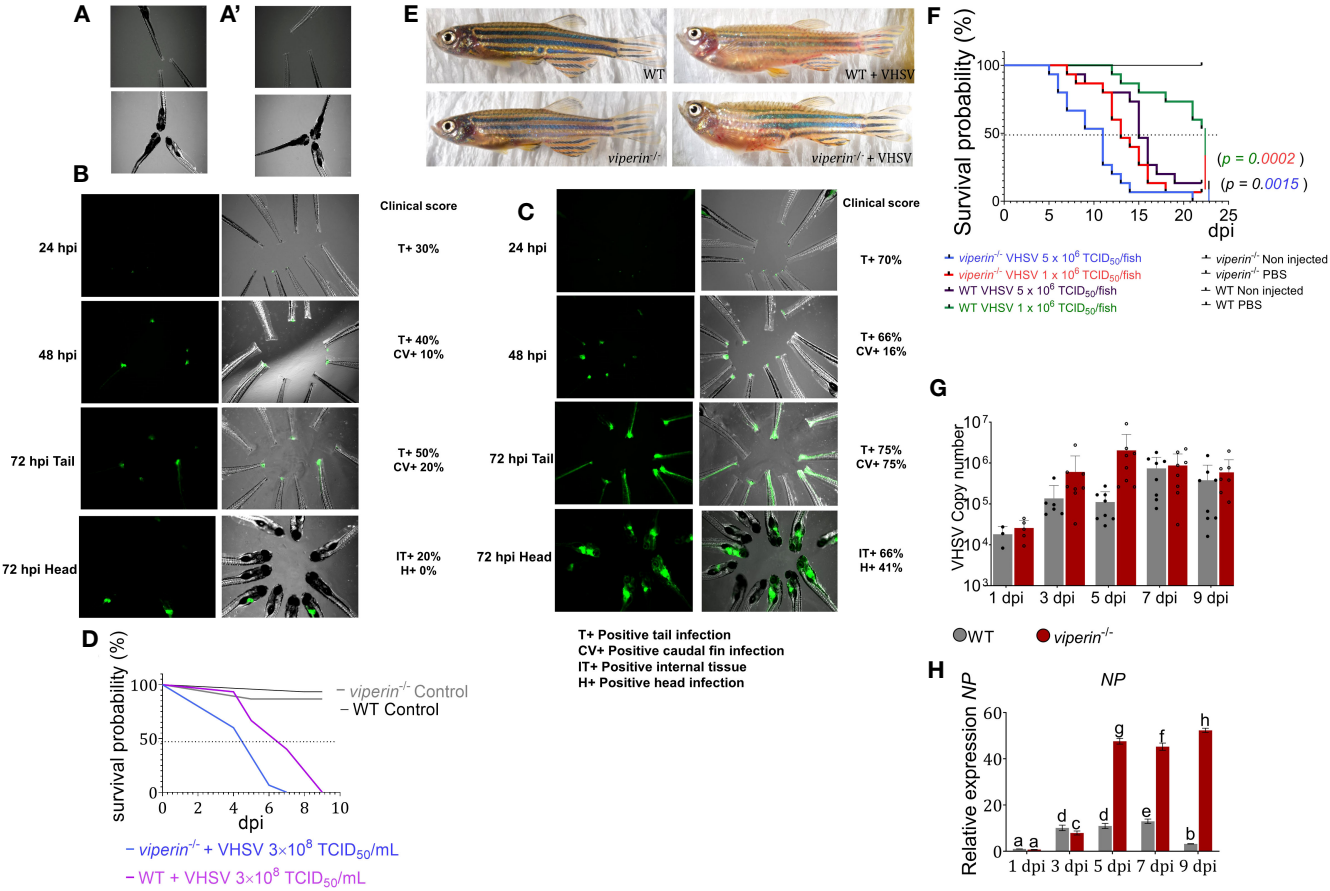
The VHSV infection experiment in *viperin* ^-/-^ zebrafish. rVHSV immersion experiment for larval zebrafish. **(A)** WT control larvae, **(A’)** *viperin* ^-/-^ control larvae. **(B)** WT, **(C)** *viperin* ^-/-^ fish were injured by caudal fin amputation method and then immersed in rVHSV. Migration of virus was analyzed after 24, 48, and 72 h postinfection (hpi). According to the experiment, rVHSV rapidly migrated into the internal tissue region of *viperin* ^-/-^ fish compared to WT fish. A clinical score represents the quantitative analysis of pathogenic symptoms. **(D)** Mortality recording for injury immersion experiment, according to the analysis, the *viperin* ^-/-^ fish had significantly higher mortality. **(E)** Morphology under VHSV infections. Adult WT and *viperin* ^-/-^ fish showed no differences in their morphology without VHSV infections. When infected with VHSV, both fish showed abnormalities such as edema, hemorrhage, and spinal defects causing head protrusions above the body axis (14 dpi). **(F)** A mortality experiment was conducted for the *viperin* ^-/-^ fish (n = 15/group). Two doses of VHSV were intraperitoneally injected into the fish (high dose 5 × 10^6^ TCID_50_/fish and the low dose 1 × 10^6^ TCID_50_/fish). Fish mortalities were recorded from 4 dpi onward. Mortality data were analyzed by the Kaplan–Meier (KM) method, and statistical significance was plotted using the Mantel–Cox test (*p* < 0.05). **(G)** VHSV copy number between the VHSV-injected *viperin* ^-/-^ and WT fish over time is shown, indicating a higher VHSV copy number in the *viperin* ^-/-^ fish. *NP* gene **(H)** expression in the *viperin* ^-/-^ fish shows significantly elevated levels compared to that in WT fish. VHSV, Viral hemorrhagic septicemia virus; rVHSV, Recombinant viral hemorrhagic septicemia virus; WT, wild type: dpi, days post infection; NP, VHSV Nucleoprotein.

In this study, the *viperin^-/-^* zebrafish model was used to understand the metabolic alterations and immune cell dynamics during VHSV infections. First, a VHSV infection procedure was developed for both adult and larval fish. For zebrafish larvae, an injury immersion experiment was conducted to understand VHSV migration from the infected site to internal tissues in *viperin^-/-^* fish. After 24 h of infections, rVHSV were observed at the sites of injury (**Figures 1A–C**). At 48 hpi, the fluorescent signals were intensified in *viperin^-/-^* compared to WT fish. At 72 hpi, VHSV migrated through the caudal vein from the injury site to the internal tissue region, and a number of *viperin^-/-^* fish had internal tissue infection at this time point compared to WT fish. A clinical score was used to compare the pathology, and the score was higher in the *viperin^-/-^* fish at all time points. A mortality analysis during the injury immersion experiment indicated higher mortality of *viperin^-/-^* compared to WT fish. VHSV infection observed at the brain region of *viperin^-/-^* may have caused significantly higher mortality compared to WT fish (**Figure 1D**).

VHSV was injected intraperitonially into adult zebrafish to evaluate survival and morphological differences (**Figure 1E**). The fish that succumbed to infection showed signs of abdominal ascites, hemorrhage, body swelling, spinal defects, externally. Internally, fish had hemorrhage in the liver, kidney, and the inner layer of the intraperitoneal cavity, and *viperin* ^-/-^ fish developed symptoms of VHSV infection more rapidly than WT fish. The daily mortality analysis indicated that *viperin* ^-/-^ zebrafish injected with a high dose of VHSV (5 × 10^6^ TCID_50_/fish) had the highest mortality rate compared to the WT fish injected with the highest dose, whereas *viperin* ^-/-^ fish injected with a lower dose of VHSV (1 × 10^6^ TCID_50_/fish) had a higher mortality rate than WT adult fish injected with the highest dose of VHSV (**Figure 1F**). VHSV copy number was analyzed (**Figure 1G**) to understand the virus proliferation. The data suggested significantly higher VHSV titers in the *viperin* ^-/-^ fish compared to those in the WT fish at 3 and 5 dpi, corroborating the higher mortality rate observed. However, in the latter part of the experiment, the VHSV copy number was quite similar between the *viperin* ^-/-^ and WT fish. The expression of the *NP* gene was significantly increased in *viperin* ^-/-^ fish at 5 dpi (**Figure 1H**; **Supplementary Figure S1**). The results of the *NP* gene expression analysis demonstrate the role of Viperin in the termination of viral gene transcription. As the main catalytic activity for Viperin suggests the ability to enervate RNA viral replication and transcription, the observed higher virus transcription and copy number indicate the effect of complete deficiency of Viperin.

### 3.2 The role of *viperin* in host metabolism during VHSV infection

**Figure 2:**
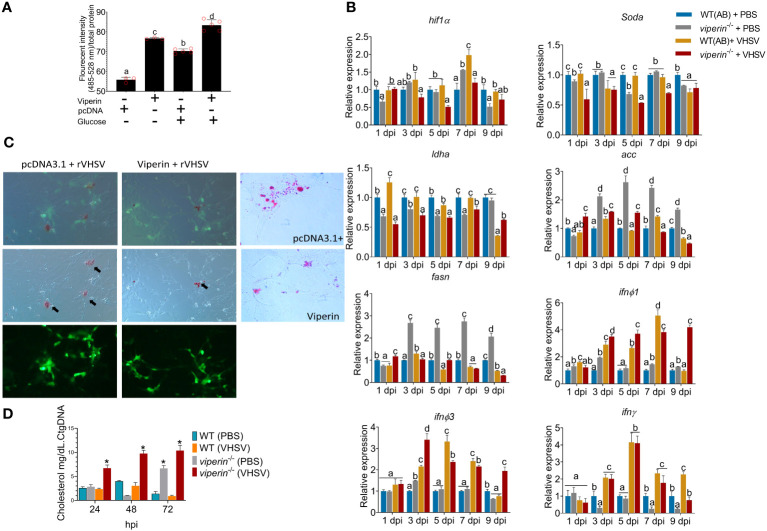
Metabolic alterations in the *viperin^-/-^* fish. **(A)** ROS quantification *in vitro*. *viperin*-overexpressing cells were analyzed for ROS production, and results are indicating a higher ROS production with the presence of Viperin. **(B)** The expression of metabolic related genes under VHSV infection in adult zebrafish. According to the analysis, the expression of *hif1a*, *soda*, and *ldha* is downregulated in *viperin* ^-/-^ fish. Expression of genes responsible for lipid synthesis, *acaca* and *fasna*, has upregulated. **(C)** Lipid analysis *in vitro*, the *viperin*-overexpressing cells had significantly lower levels of lipids under virus infection. **(D)** Cholesterol analysis *in vivo*, *viperin* ^-/-^ fish had a significantly higher amount of cholesterol compared to that in WT fish. ROS, Reactive oxygen species; hif1a, hypoxia-inducible factor-1; soda, superoxide dismutase; ldha, Lactate dehydrogenase; acc, Acetyl-CoA carboxylase; fasn, fatty acid synthase; ifn, interferon.

In this section, the involvement of Viperin in host metabolism during VHSV infection was evaluated. First, ROS production under *viperin* overexpression was analyzed in ZF4 cells (**Figure 2A**). For this experiment, glucose was externally supplemented to cells to increase the rate of metabolism. Interestingly, *viperin* overexpression resulted in a higher ROS generation even without glucose supplementation. When glucose is supplemented, control cells enhanced the ROS production and the *viperin*-overexpressing line had significantly higher ROS generation.

Metabolic genes were compared during VHSV infection in *viperin* ^-/-^ to WT *in vivo* to understand the relative differences in metabolic related gene expression under Viperin deficiency. The expression of *hypoxia-inducible factor*-1 (*hif1α*), superoxide dismutase-a (*soda*), and lactate dehydrogenase-A (*ldha*) had a relatively lower expression in the *viperin^-/-^* fish compared to WT (**Figure 2B**). Elevated ROS can trigger the *hif1a* promoter leading to its expression (17). The reduced ROS in fish due to the lack of Viperin may have influenced the observed reduction of *hif1α* and *soda.* As the expression of *ldha* is regulated by the Hif1α (18), the observed downregulation of *hif1α* may have resulted in the observed reduction of the *ldha*.

Genes responsible for lipid synthesis, fatty acid synthase (*fasna*) and acetyl-CoA carboxylase (*acc*), indicated a significant upregulation in *viperin* ^-/-^ fish (**Figure 2B**). Host lipids play an important role for the enveloped virus egress (19). Studies indicated that cells could reduce lipid synthesis during viral infections as an antiviral strategy. Moreover, drugs that inhibit lipid synthesis such as lovastatin bear antiviral properties (19, 20). *In vitro* lipid analysis revealed a considerable reduction under *viperin* overexpression (**Figure 2C**). Then, lipids were analyzed under VHSV infections. In control cells, a higher lipid synthesis was observed during VHSV infection. When *viperin* is overexpressed, lipids were considerably reduced. Cholesterol analysis *in vivo* (**Figure 2D**) indicated a significantly higher cholesterol production in *viperin* ^-/-^ fish during VHSV infection. In the absence of *viperin*, enhanced lipid and lower ROS levels may negatively affect antiviral defense.

### 3.3 Neutrophil and macrophage dynamics in *viperin* ^-/-^ fish

**Figure 3:**
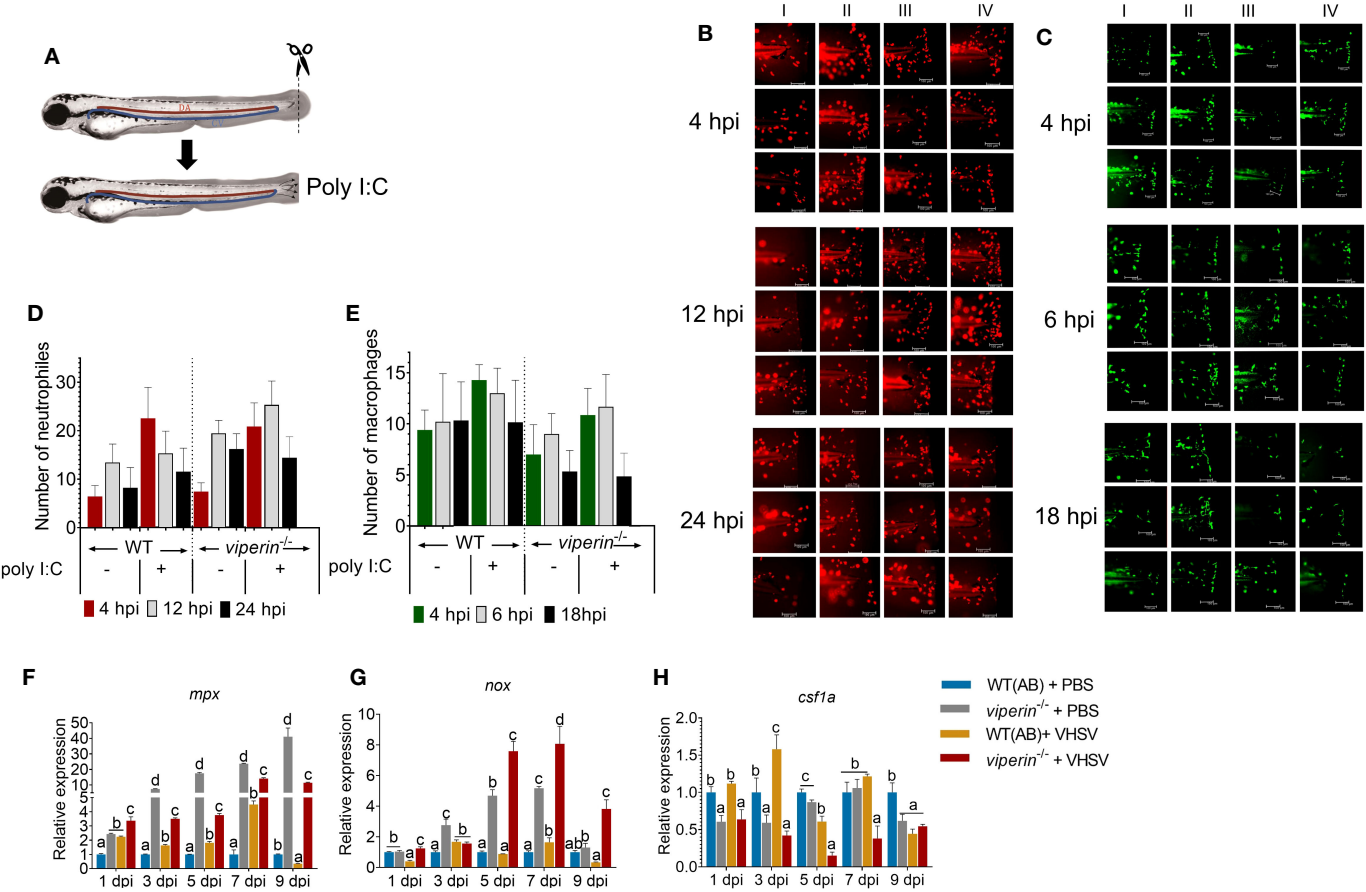
Neutrophil and macrophage recruitment in *viperin* ^-/-^ fish. **(A)** Zebrafish lines *viperin* ^-/-^ *Tg*(*mpx:mcherry*) and *viperin* ^-/-^ *Tg(mpeg:egfp)* were generated to track neutrophils and macrophages, respectively. The caudal fin was amputated of these lines and immersed in poly I:C. **(B)** Analysis of neutrophil recruitment, **(C)** the recruitment of the macrophages to the site of infection was observed in a time-dependent manner (I-Wt PBS, II-Wt+Poly I:C, III-*viperin* ^-/-^ + PBS, and IV-*viperin* ^-/-^ + Poly I:C). Dynamics of the neutrophils **(D)**, macrophages **(E)** at the wound site showed time-dependent differences between the WT and *viperin* ^-/-^ fish. Expression analysis of neutrophil marker genes *mpx* **(F)** and *nox* **(G)** in the adult zebrafish with VHSV infection showed significant upregulation of the marker genes in *viperin* ^-/-^ fish compared to those in WT fish. Gene expression analysis for the marker gene *csf1a* **(H)** for macrophages, indicating significant downregulation in *viperin* ^-/-^ fish. Poly I:C, Polyinosinic:polycytidylic acid; hpi, Hours post infection; mpx, myeloid-specific peroxidase; nox, NADPH oxidase; csf1a, colony stimulating factor 1.

Metabolic alterations may interfere with immune cell dynamics during virus infections (21). We generated *viperin* ^-/-^ *Tg*(*mpx:mCherry*) and *viperin* ^-/-^ *Tg*(*mpeg:EGFP*) (**Figure 3A**) models to evaluate neutrophil and macrophage dynamics, respectively. The count of neutrophils at the site of infection was higher in the *viperin* ^-/-^ fish than that in WT fish. Even though, in the WT fish, the neutrophil count was enhanced and dropped quickly at the site of infection, comparatively a higher number of neutrophils were retained in the *viperin* ^-/-^ fish (**Figures 3B, D**). This may suggest a higher neutrophil survival in the *viperin^-/-^* fish than that in WT fish.

An opposite observation was made with the number of macrophages recruited to the site of infection, where the number of macrophages was significantly reduced in *viperin* ^-/-^ fish compared to that in WT fish (**Figures 3C, E**). During the inflammatory M1 activation, pentose phosphate pathway (PPP) activation was shown in macrophages like neutrophils (22). Even though both macrophages and neutrophils are phagocytic cells with similar origins, there are some notable differences. One major difference is that the production of ROS is more prominent in neutrophils than that in macrophages (23). This may explain the short life spans of neutrophils. Therefore, the effect of Viperin may be directly observable in neutrophils compared to macrophages. Additionally, there is evidence that macrophages will aid wound-healing processes after a pathogen threat is resolved in wound (24). However, in *viperin* ^-/-^ fish, the entire injury was not covered by macrophages, which may indicate a negative association with the subsequent wound-healing process. In WT fish, with or without immune stimulation, macrophages were aligned at the edge of the wound, creating an effective defense barrier.

Neutrophil- and macrophage-specific marker expression was analyzed to determine the impact of Viperin on the observed results. Significant upregulation of *mpx* in *viperin* ^-/-^ fish was observed (**Figure 3F**). The expression analysis of *nox1* (**Figure 3G**) resembled the expression pattern of the *mpx* gene. To confirm the results of macrophage recruitment to the wound site, we analyzed macrophage marker *csf1a* (**Figure 3H**). The expression of *csf1a* was reduced in the *viperin* ^-/-^ model, confirming the data for macrophage migration.

## 4 Discussion

The inhibitory nucleotide produced by Viperin, ddhCTP, binds to RdRp of VHSV, inhibiting viral gene expression and genomic replication. Hence, elevated virus gene expression and VHSV copy numbers are present in *viperin* ^-/-^ fish without ddhCTP interference (3). Apart from this, ddhCTP could alter host metabolism under virus infection. Viruses facilitate their replication and propagation through modifying host cellular metabolism (25). For instance, viruses may convert pyruvate into lactate-by-lactate dehydrogenase-A (Ldha) rather than pyruvate consumed in the TCA cycle or electron transport chain. This phenomenon is called the Warburg effect, and its primary role is to reduce glucose to a molecule with more carbon atoms, allowing carbon biomass accumulation in infected cells (26). This carbon accumulation eventually facilitates an increase in viral biomass (27).

When the viral Pathogen-associated molecular patterns (PAMs) are recognized by either a surface or an internal receptor (28), the cellular pyruvate yield is reduced, and the NADPH yield is significantly enhanced through the PPP. As both glycolysis and PPP are dependent upon glucose, logically, an inhibition of glycolysis pathway may spare glucose pools to divert through PPP. ddhCTP was shown to inhibit GAPDH, which is a major regulatory protein in the cellular glycolysis pathway. Inhibition of GAPDH may cause glucose to accumulate inside the cells and be used by PPP and resulting in NADPH generation, which could participate in ROS generation through Nox2 complex. Even though this mechanism could explain the higher ROS production, a recent study indicated that ddhCTP is unable to interfere with GAPDH or LDH as suggested by Ebrahimi et al. (29). Therefore, the clues for the Viperin’s activity on metabolism could be far more complicated and indirect, and more studies may be required to explain the real relationship between Viperin and ROS.

Lipids are especially important for enveloped viruses to proceed through their life cycles, including human cytomegalovirus (HCMV), resulting in increased lipid synthesis under infections (30). In this study, they suggested that Viperin inhibits β-oxidation; hence, a higher lipid accumulation was observed in the cells. Acetyl-CoA carboxylase-α (Accα) and fatty acid synthase (Fasn) are two regulatory enzymes for the fatty acid synthesis pathway. The significant upregulation of the lipid synthesis genes observed in this study may relate to the elevated β-oxidation rates under the Viperin deficiency. Several viral infections have been reported to induce *acc* and *fasn* upregulation. At the site of VHSV infection, an elevated lipid production was observed in the present study, this observation agrees with several previous observations (31). However, mitochondrial dysfunction during the pathogenic infections as well as the energy required for the *viperin* overexpression could be a potential limitation for explaining observations in the present study.

VHSV is an enveloped virus, highlighting the potential importance of host cellular lipids in its propagation cycle, especially for the virus egress process, cell membrane integrity is vital. Whereas the plasma membrane cholesterols are a key component for membrane integrity and fluidity (32). If the cholesterol ratio in the cell membrane changes, virus release will be impaired (6). Viruses alter cholesterol biosynthesis by inhibiting activated protein kinase (AMPK) (33). ddhCTP, produced by Viperin, can directly inhibit several genes involved in the cholesterol biosynthesis pathway including farnesyl pyrophosphate synthase (FPPS), reducing cholesterol in cells (7).

Expression of *viperin* is highly induced in neutrophils and macrophages during virus infections (34). For both cell populations, oxidative activation is critical for their immune role. A deficiency in ROS production in phagocytes caused diseases such as chronic granulomatous disease (CGD), in which immune cells show a delayed phagocytosis activity (35). These diseases indicated the importance of the proper generation of ROS for rapid clearance of pathogens. Immune cells at their resting state undergo glycolysis for their energy demands and for oxidative phosphorylation. The reduced ROS production observed may trigger the *mpx* expression in *viperin* ^-/-^ fish. The neutrophil activation process is ROS-dependent (36), and the higher ROS in the neutrophils in the WT fish might reduce the neutrophil lifetime compared to that in the *viperin* ^-/-^ fish. Our result suggests that lacking Viperin may not interfere with the neutrophil migration process. But the enhanced lifetime of neutrophils in *viperin* ^-/-^ fish may explain the higher count at the site of infection. However, the exact cause of higher neutrophil counts at the wound site requires further investigation. In murine models, Viperin interferes with macrophage polarization events (37). Studies in mouse models have revealed an interconnection between macrophages and host cellular metabolism. Activation of PPP is a crucial determinant of proper immune reactivity and macrophage polarization (38).

In summary, the relevance of zebrafish Viperin in virus-induced metabolic alterations was analyzed in this study. *In vivo*, virus challenge experiments indicated the importance of Viperin for fish survival, whereas *viperin* ^-/-^ fish had significantly lower survival rates and higher VHSV titers. The *viperin* overexpression experiments suggested significant alteration to lipid and ROS production both *in vitro* and *in vivo*. Macrophage recruitment to the site of infection was reduced, and the pattern of neutrophil recruitment was different from that in the WT fish.

Host cellular metabolism works as a complex network; the result of this study demonstrates the role of Viperin in interfering with lipid metabolism and ROS in zebrafish. These alterations appeared in the activation and the function of the immune cells such as neutrophils and macrophages, thereby altering the host–pathogen landscape. Taken together, this study showed Viperin as an important regulator of metabolism under virus infections, and the activity may be important for host defense. Finally, the therapeutic usage of the product of Viperin, ddhCTP, may be vital in translational research.

## Data availability statement

The data presented in the study are deposited in the NCBI GEO repository, accession number GSE250036.

## Ethics statement

The animal study was approved by The Jeju National University Animal Ethics Committee. The study was conducted in accordance with the local legislation and institutional requirements.

## Author contributions

KS: Conceptualization, Data curation, Formal analysis, Methodology, Visualization, Writing – original draft, Software. SJ: Conceptualization, Formal analysis, Investigation, Methodology, Resources, Supervision, Writing – review & editing. KM: Conceptualization, Formal analysis, Methodology, Software, Visualization, Writing – original draft. M-JK: Conceptualization, Formal analysis, Investigation, Methodology, Validation, Visualization, Writing – review & editing. JL: Conceptualization, Data curation, Formal analysis, Funding acquisition, Investigation, Methodology, Project administration, Resources, Software, Supervision, Validation, Visualization, Writing – review & editing.

## References

[1] Liu S Li N Lin Q Liu L Niu Y Liang H. Glutaminase 1 in mandarin fish Siniperca chuatsi: Molecular characterization, expression pattern and function involving in virus replication. Aquaculture (2020) 519:734924. doi: 10.1016/j.aquaculture.2020.734924

[2] Girdhar K Powis A Raisingani A Chrudinová M Huang R Tran T. Viruses and metabolism: the effects of viral infections and viral insulins on host metabolism. Annu Rev Virol (2021) 8:373–91. doi: 10.1146/annurev-virology-091919-102416 PMC9175272 34586876

[3] Rivera-Serrano EE Gizzi AS Arnold JJ Grove TL Almo SC Cameron CE. Viperin reveals its true function. Annu Rev Virol (2020) 7:421–46. doi: 10.1146/annurev-virology-011720-095930 PMC8191541 32603630

[4] Palmer CS. Innate metabolic responses against viral infections. Nat Metab (2022) 4:1245–59. doi: 10.1038/s42255-022-00652-3 36266542

[5] Honarmand Ebrahimi K Vowles J Browne C Mccullagh J James WS. ddhCTP produced by the radical-SAM activity of RSAD2 (viperin) inhibits the NAD+-dependent activity of enzymes to modulate metabolism. FEBS Lett (2020) 594:1631–44. doi: 10.1002/1873-3468.13778 32232843

[6] Dai J Wang H Liao Y Tan L Sun Y Song C. Coronavirus infection and cholesterol metabolism. Front Immunol (2022) 13:791267. doi: 10.3389/fimmu.2022.791267 35529872 PMC9069556

[7] Grunkemeyer TJ Ghosh S Patel AM Sajja K Windak J Basrur V. The antiviral enzyme viperin inhibits cholesterol biosynthesis. J Biol Chem (2021) 297:100824. doi: 10.1016/j.jbc.2021.100824 34029588 PMC8254119

[8] Baillon L Merour E Cabon J Louboutin L Vigouroux E Alencar ALF. The viral hemorrhagic septicemia virus (VHSV) markers of virulence in rainbow trout (Oncorhynchus mykiss). Front Microbiol (2020) 11:574231. doi: 10.3389/fmicb.2020.574231 33193184 PMC7606196

[9] Zhang W Yao L Chen X Li M Yi M Jia K. Functional characterization of myosin III light chain b of sea perch (Lateolabrax japonicus) in response to fish virus infection. Aquaculture (2022) 550:737840. doi: 10.1016/j.aquaculture.2021.737840

[10] Adamek M Davies J Beck A Jordan L Becker AM Mojzesz M. Antiviral actions of 25-hydroxycholesterol in fish vary with the virus-host combination. Front Immunol (2021) 12:581786. doi: 10.3389/fimmu.2021.581786 33717065 PMC7943847

[11] Shanaka K Jung S Madushani KP Wijerathna H Neranjan Tharuka MD Kim MJ. Generation of viperin-knockout zebrafish by CRISPR/Cas9-mediated genome engineering and the effect of this mutation under VHSV infection. Fish Shellfish Immunol (2022) 131:672–81. doi: 10.1016/j.fsi.2022.10.040 36309322

[12] Avdesh A Chen M Martin-Iverson MT Mondal A Ong D Rainey-Smith S. Regular care and maintenance of a zebrafish (Danio rerio) laboratory: an introduction. J Vis Exp (2012):e4196. doi: 10.3791/4196 23183629 PMC3916945

[13] Kim JO Kim WS Kim SW Han HJ Kim JW Park MA. Development and application of quantitative detection method for viral hemorrhagic septicemia virus (VHSV) genogroup IVa. Viruses (2014) 6:2204–13. doi: 10.3390/v6052204 PMC4036551 24859343

[14] Bustin SA Benes V Garson JA Hellemans J Huggett J Kubista M. The MIQE guidelines: minimum information for publication of quantitative real-time PCR experiments. Clin Chem (2009) 55:611–22. doi: 10.1373/clinchem.2008.112797 19246619

[15] Madushani KP Shanaka K Jung S Kim MJ Lee J. Ablation of myd88 alters the immune gene expression and immune cell recruitment during VHSV infection in zebrafish. Fish Shellfish Immunol (2023) 141:109006. doi: 10.1016/j.fsi.2023.109006 37598733

[16] Kim MS Kim DS Kim KH. Generation and characterization of NV gene-knockout recombinant viral hemorrhagic septicemia virus (VHSV) genotype IVa. Dis Aquat Organ (2011) 97:25–35. doi: 10.3354/dao02394 22235592

[17] Bonello S Zahringer C Belaiba RS Djordjevic T Hess J Michiels C. Reactive oxygen species activate the HIF-1alpha promoter *via* a functional NFkappaB site. Arterioscler Thromb Vasc Biol (2007) 27:755–61. doi: 10.1161/01.ATV.0000258979.92828.bc 17272744

[18] Pathria G Scott DA Feng Y Sang Lee J Fujita Y Zhang G. Targeting the Warburg effect *via* LDHA inhibition engages ATF4 signaling for cancer cell survival. EMBO J (2018) 37. doi: 10.15252/embj.201899735 PMC6187221 30209241

[19] Chu J Xing C Du Y Duan T Liu S Zhang P. Pharmacological inhibition of fatty acid synthesis blocks SARS-CoV-2 replication. Nat Metab (2021) 3:1466–75. doi: 10.1038/s42255-021-00479-4 PMC8475461 34580494

[20] Farfan-Morales CN Cordero-Rivera CD Reyes-Ruiz JM Hurtado-Monzon AM Osuna-Ramos JF Gonzalez-Gonzalez AM. Anti-flavivirus properties of lipid-lowering drugs. Front Physiol (2021) 12:749770. doi: 10.3389/fphys.2021.749770 34690817 PMC8529048

[21] Jung J Zeng H Horng T. Metabolism as a guiding force for immunity. Nat Cell Biol (2019) 21:85–93. doi: 10.1038/s41556-018-0217-x 30602764

[22] Thapa B Lee K. Metabolic influence on macrophage polarization and pathogenesis. BMB Rep (2019) 52:360–72. doi: 10.5483/BMBRep.2019.52.6.140 PMC6605523 31186085

[23] Silva MT. When two is better than one: macrophages and neutrophils work in concert in innate immunity as complementary and cooperative partners of a myeloid phagocyte system. J Leukoc Biol (2010) 87:93–106. doi: 10.1189/jlb.0809549 20052802

[24] Krzyszczyk P Schloss R Palmer A Berthiaume F. The role of macrophages in acute and chronic wound healing and interventions to promote pro-wound healing phenotypes. Front Physiol (2018) 9:419. doi: 10.3389/fphys.2018.00419 29765329 PMC5938667

[25] Varanasi SK Rouse BT. How host metabolism impacts on virus pathogenesis. Curr Opin Virol (2018) 28:37–42. doi: 10.1016/j.coviro.2017.11.003 29156318

[26] Vander Heiden MG Cantley LC Thompson CB. Understanding the Warburg effect: the metabolic requirements of cell proliferation. Science (2009) 324:1029–33. doi: 10.1126/science.1160809 PMC2849637 19460998

[27] Sumbria D Berber E Mathayan M Rouse BT. Virus infections and host metabolism-can we manage the interactions? Front Immunol (2020) 11:594963. doi: 10.3389/fimmu.2020.594963 33613518 PMC7887310

[28] Li W Wang H Zheng SJ. Roles of RNA sensors in host innate response to influenza virus and coronavirus infections. Int J Mol Sci (2022) 23. doi: 10.3390/ijms23158285 PMC9368391 35955436

[29] Lee JH Wood JM Almo SC Evans GB Harris LD Grove TL. Chemoenzymatic synthesis of 3'-deoxy-3',4'-didehydro-cytidine triphosphate (ddhCTP). ACS Bio Med Chem Au (2023) 3:322–6. doi: 10.1021/acsbiomedchemau.3c00014 PMC10436258 37599790

[30] Seo JY Cresswell P. Viperin regulates cellular lipid metabolism during human cytomegalovirus infection. PloS Pathog (2013) 9:e1003497. doi: 10.1371/journal.ppat.1003497 23935494 PMC3731232

[31] Liu YY Liang XD Liu CC Cheng Y Chen H Baloch AS. Fatty acid synthase is involved in classical swine fever virus replication by interaction with NS4B. J Virol (2021) 95:e0078121. doi: 10.1128/JVI.00781-21 34132567 PMC8354233

[32] Yang S-T Kreutzberger AJ Lee J Kiessling V Tamm LK. The role of cholesterol in membrane fusion. Chem Phys Lipids (2016) 199:136–43. doi: 10.1016/j.chemphyslip.2016.05.003 PMC4972649 27179407

[33] Ran C Xie M Li J Xie Y Ding Q Li Y. Dietary nucleotides alleviate hepatic lipid deposition *via* exogenous AMP-mediated AMPK activation in zebrafish. J Nutr (2021) 151:2986–96. doi: 10.1093/jn/nxab232 34383941

[34] Hinson ER Joshi NS Chen JH Rahner C Jung YW Wang X. Viperin is highly induced in neutrophils and macrophages during acute and chronic lymphocytic choriomeningitis virus infection. J Immunol (2010) 184:5723–31. doi: 10.4049/jimmunol.0903752 PMC3883313 20410488

[35] Yu HH Yang YH Chiang BL. Chronic granulomatous disease: a comprehensive review. Clin Rev Allergy Immunol (2021) 61:101–13. doi: 10.1007/s12016-020-08800-x 32524254

[36] Wang Z Lin L Chen W Zheng X Zhang Y Liu Q. Neutrophil plays critical role during Edwardsiella piscicida immersion infection in zebrafish larvae. Fish Shellfish Immunol (2019) 87:565–72. doi: 10.1016/j.fsi.2019.02.008 30742890

[37] Eom J Yoo J Kim JJ Lee JB Choi W Park CG. Viperin deficiency promotes polarization of macrophages and secretion of M1 and M2 cytokines. Immune Netw (2018) 18:e32. doi: 10.4110/in.2018.18.e32 30181920 PMC6117515

[38] Nagy C Haschemi A. Time and demand are two critical dimensions of immunometabolism: the process of macrophage activation and the pentose phosphate pathway. Front Immunol (2015) 6:164. doi: 10.3389/fimmu.2015.00164 25904920 PMC4389563

