# Supplementary material for: Viperin mutation is linked to immunity, immune cell dynamics, and metabolic alteration during VHSV infection in zebrafish
Source: Front Immunol. 2023 Dec 19;14:1327749. doi: 10.3389/fimmu.2023.1327749 (PMC10763233; doi:10.3389/fimmu.2023.1327749)
Supplement: Supplementary file 2 [file DataSheet_2.docx]

Supplementary Material

# Tables

**Supplementary Table 1**-Primes used in this study.

| Gene | Primer efficiency (%) | Amplicon size | Forward and reverse Primer (5’-3’) |
| --- | --- | --- | --- |
| VHSV *NP* gene (AC: AGS83377) | 98 | 104 | TGTCTCAGATCAGTGGGAAGTACGC |
|  |  |  | GGACCTCAGCGACAAGTTCGG |
| *viperin* | 95 | 102 | CTGGTGTCGAGAGTATAAGGTGGCTTTC |
|  |  |  | TACTGGATTGAGAGCGGTGATCTGC |
| *soda* | 98 | 105 | AACATGGTTTCCACGTCCATGC |
|  |  |  | TATCGGTTGGCCCACCATGAGTTT |
| *hif1a* | 96 | 108 | AGCAGATCCTGTTCTCACGCT |
|  |  |  | AGGACATCTGAAGAGGGCAACATGAC |
| *ldha* | 100 | 99 | GGTTGGGTCGTTGGAGAACATGG |
|  |  |  | TACCCAAGTCAGGGTTTAGAGCTTGG |
| *fasna* | 95 | 119 | ACGGCAATGTCACCCTACTGAGAGC |
|  |  |  | GCCTTCGATCACATGAACTGACACCTTCC |
| *acaca* | 95 | 100 | AGGCGCCAGATCCGTCATCG |
|  |  |  | GAGTCCATAGCCACCTCAGGGTTAGC |
| *ifnφ1* | 96 | 103 | GCCAGGGAGCACATGAACTCGG |
|  |  |  | TTGCCACACATTCTTTGAGG |
| *ifnφ3* | 96 | 90 | GAGAACTTCGGTGATCCGGACC |
|  |  |  | CATGATGCATGTGCTGTATTTAATCTGCCG |
| *ifnγ* | 94 | 95 | AGAGCTCAGGACGTATGCAGAAACG |
|  |  |  | TATAGACACGCTTCAGCTCAAACAAAGCC |
| *mpx* | 98 | 120 | CTGAACCCTGCTTCCCAATTTCG |
|  |  |  | CTTCACCAAACATGTAGGCAGTGTGC |
| *nox1* | 96 | 102 | TTTAGGGTCTGCATTGGCTTGGG |
|  |  |  | GCAGCAGAGACAGCAGGTTACG |
| *csf1a* | 99 | 103 | GAGTCCGATGAGGGTGCCAAG |
|  |  |  | CGTCTGGGTCAGTCTGGTCCTTTATC |
| *viperin* cloning primers | NA | 1107 | GAGAGAaagcttACCATGGTGACATCAAACCAACTTGG (HindIII) |
|  |  |  | GAGAGActcgagTCACCATTCCAGTTTCATATCTGC (XhoI) |

# Supplementary Figures


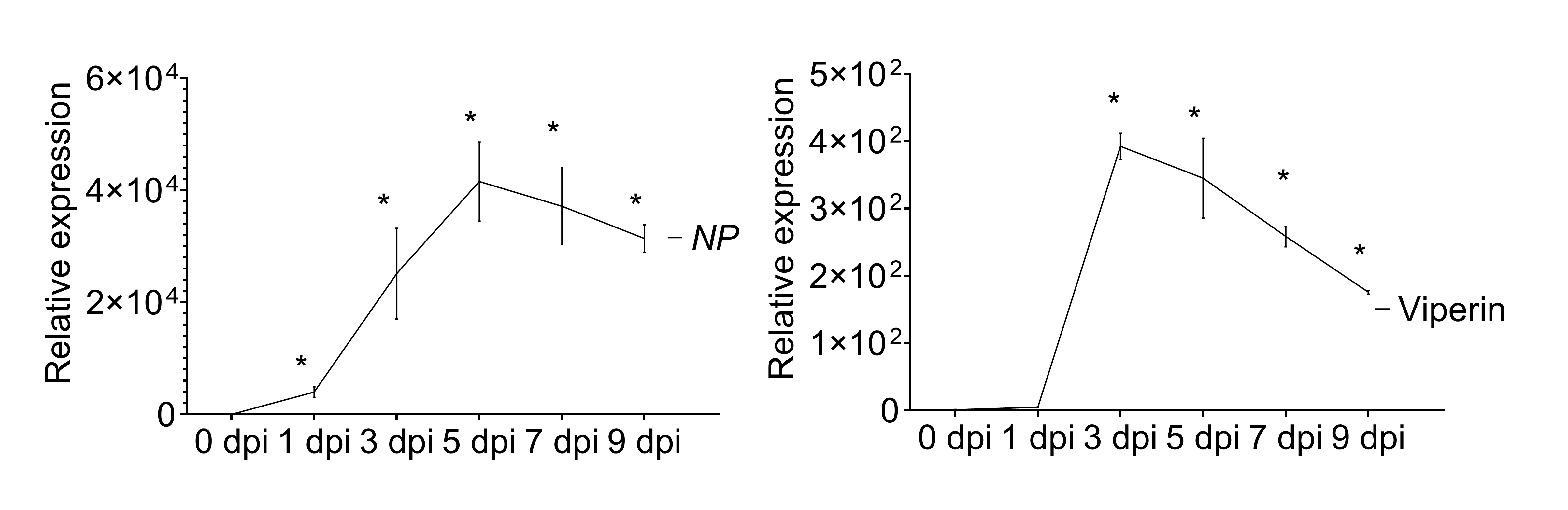


**Supplementary Figure 1.** Expressional variation of VHSV NP gene vs viperin gene in the VHSV infected Wt fish.
